# Brazilin Inhibits the Proliferation of Non‐Small Cell Lung Cancer by Regulating the STING/TBK1/IRF3 Pathway

**DOI:** 10.1111/jcmm.70688

**Published:** 2025-07-01

**Authors:** Li‐Ping Kang, Cong Xu, Pan Xu, Dong‐Hui Huang, Ze‐Bo Jiang

**Affiliations:** ^1^ Zhuhai Hospital of Integrated Traditional Chinese & Western Medicine Zhuhai Guangdong China; ^2^ State Key Laboratory of Quality Research in Chinese Medicine Macau University of Science and Technology Macao Taipa Macau (SAR) China

**Keywords:** brazilin, CXCL10, non‐small cell lung cancer, STING

## Abstract

Non‐small cell lung cancer (NSCLC) stands as a predominant cause of cancer‐related mortality worldwide. Brazilin, an active isoflavonoid compound derived from Chinese herbs, has displayed anti‐cancer properties across various cancer cell lines. However, the precise anti‐tumour mechanism of Brazilin in NSCLC remains incompletely understood. In this paper, we demonstrated that Brazilin treatment significantly reduced the proliferation of NSCLC cells and induced apoptosis. Additionally, Brazilin caused G2 cell cycle arrest in NSCLC cells, characterised by decreased expression of Cyclin B1 and increased expression of P21. Brazilin also induced mitochondrial dysfunction and ROS production in NSCLC cells. Mechanistically, Brazilin treatment significantly activated the STING pathway and upregulated the expression of CXCL10, CXCL9, and CCL5 in NSCLC cell lines. Notably, the inhibition of the STING pathway with H‐151 enhances cell viability, suggesting STING is involved in Brazilin‐induced apoptosis. These findings underscore Brazilin as a promising anti‐cancer agent for NSCLC.

**Abbreviations**
CCK8: cell counting kit‐8
CCL5: C‐C motif chemokine ligand 5
CXCL10: C‐X‐C motif chemokine ligand 10
CXCL9: C‐X‐C motif chemokine ligand 9
DMSO: dimethyl sulfoxide
FBS: fetal bovine serum
GAPDH: glyceraldehyde‐3‐phosphate dehydrogenase
IRF3: interferon regulatory factor 3
NSCLC: non‐small cell lung cancer
PD‐L1: programmed cell death ligand 1
PFA: paraformaldehyde
PI: propidium iodide
ROS: reactive oxygen species
STING: stimulator of interferon genes
TBK1: TANK‐binding kinase 1

## 1 Introduction

Lung cancer ranks as the primary cause of cancer‐related mortality globally, with non‐small cell lung cancer (NSCLC) representing the most prevalent histological subtype, comprising about 80%–85% of all lung cancer cases [1, 2]. Despite the availability of various treatments, including surgery, radiotherapy, chemotherapy, and immunotherapy, survival rates for advanced stage NSCLC patients remain dismally low [3]. Chemotherapy often results in severe side effects and the development of drug resistance, prompting a shift towards targeted therapies. Unfortunately, tumours can also become resistant to these targeted treatments over time, diminishing their efficacy [4, 5].

The stimulator of interferon genes (STING) signalling pathway is pivotal in cancer therapy, as it enhances the immune response to tumour‐associated nucleic acids [6]. Upon detecting cytosolic DNA, STING activates TANK‐binding kinase 1 (TBK1), which subsequently phosphorylates Interferon Regulatory Factor 3 (IRF3) [7]. This cascade leads to the production of type I interferons and other pro‐inflammatory cytokines, fostering an inflammatory tumour microenvironment that invites and activates immune cells [8]. The significance of the STING pathway in cancer therapy lies in its capacity to bolster anti‐tumour immunity. By activating STING, therapeutic strategies can elicit systemic immune responses that target and eradicate cancer cells [9]. Moreover, STING agonists are being investigated as adjuvants in combination with other therapies, such as checkpoint inhibitors and chemotherapy, to counteract the immune evasion strategies employed by tumours [10]. The STING/TBK1/IRF3 pathway's role in modulating tumour microenvironments may also help alleviate immune suppression, positioning it as a critical target for novel cancer treatments that leverage the body's immune system for enhanced efficacy against malignancies.

Brazilin, a natural plant isoflavone found in plants such as *Caesalpinia sappan* and *Haematoxylum braziletto*, has garnered attention for its potential medicinal properties, including anti‐inflammatory effects relevant to various conditions like fever, bleeding, rheumatism, skin disorders, diabetes, and cardiovascular diseases [11]. Brazilin has demonstrated anticancer properties across multiple cancer types, including ovarian, colorectal, gastric, pancreatic, and breast cancers [12]. Its mechanisms of action include disrupting cell signalling pathways, inducing cell cycle arrest, promoting apoptosis, modulating DNMT1 expression, and inhibiting tumour angiogenesis [13]. However, the specific anticancer potential and mechanisms of Brazilin on the context of NSCLC remain underexplored.

In this study, we examine the impact of Brazilin on cell viability, cell cycle progression, and apoptosis in human NSCLC cell lines to unravel its potential mechanisms. Our findings indicate that Brazilin hinders the growth of NSCLC cells by inducing G2/M phase arrest and subsequent apoptosis. Additionally, Brazilin induced mitochondrial dysfunction and ROS production and activated the STING/TBK1/IRF3 pathway. Of note, Brazilin prominently activates the STING pathway in NSCLC, triggering the expression of C‐X‐C motif chemokine ligand 10 (CXCL10), C‐X‐C Motif Chemokine Ligand 9 (CXCL9) and C‐C motif chemokine ligand 5 (CCL5), potentially assisting in overcoming immunotherapy resistance.

## 2 Materials and Methods

### 2.1 Reagents

Brazilin (purity ≥ 98%, # S9428) was sourced from Selleck Chemicals (Houston, TX, USA), while N‐acetyl‐L‐cysteine (NAC, #A105422) was obtained from Aladdin (Guangzhou, China). The BCA protein detection kit (#P0010), RIPA lysis buffer (#P0013C), and SDS‐PAGE protein sample loading buffer (#P0015L) were purchased from Shanghai Fengtai Biotechnology Co. Ltd. STING antagonist (H‐151) (#S6652) was acquired from Selleck Chemicals (Houston, TX, USA). Antibodies for Phospho‐STING (CST#50907), Phospho‐TBK1/NAK, TBK1 (CST#38066), IRF3 (CST#11904), Phospho‐IRF‐3 (CST#4947), Glyceraldehyde‐3‐phosphate dehydrogenase (GAPDH) and STING (CST#13647) were obtained from Cell Signalling Technology (Danvers, MA, USA). Fluorescein‐conjugated goat anti‐rabbit and anti‐mouse secondary antibodies were procured from Odyssey (Belfast, ME, USA). The Annexin V/PI staining dye was obtained from BD Biosciences (San Jose, CA, USA).

### 2.2 Cell Line and Cell Culture

Human NSCLC cell lines A549 and H358 were obtained from Macau University of Science and Technology. These cells were cultured in Roswell Park Memorial Institute (RPMI)‐1640 medium, supplemented with 10% fetal bovine serum (FBS, 10270–106, Gibco, USA) and 1% penicillin‐streptomycin solution (15140–122, Gibco, USA). All cultures were maintained in a controlled environment at 37°C with 5% CO_2_.

### 2.3 Cell Viability

Cell viability was assessed as previously reported [14]. A549 and H358 cells were selected in optimal condition, and their concentrations were adjusted to evaluate cell proliferation. Cells were seeded into 96‐well plates at a density of 3000 cells per well and incubated for 24, 48 and 72 h. Cell viability was assessed using the Cell Counting Kit‐8 (#CK04‐500 T, Dojindo, Japan). After incubation, Cell Counting Kit‐8 (CCK8) solution was added, and the cells were incubated for an additional 4 h at 37°C. Absorbance was then measured at 450 nm using microplate reader (Beckman, USA).

### 2.4 Cell Apoptosis

To evaluate the effect of Brazilin on apoptosis in NSCLC cells, we adhered to the previously published protocol [15]. Cells were treated with Brazilin for 24 h, then digested, washed with PBS, and stained using the BD Annexin V/PI double staining kit (#556547, BD Biosciences, USA). After a 30‐min dark incubation cells were processed as per the kit instructions. Briefly, harvested cells were washed with PBS and stained with Annexin V‐FITC and propidium iodide (PI). Flow cytometry was employed to analyse the stained cells and quantify the percentage of apoptotic cells, including early apoptotic cells (Annexin V positive, PI negative) and late apoptotic/necrotic cells (Annexin V positive, PI positive).

### 2.5 Colony Formation

Colony formation assays were conducted to evaluate the effect of Brazilin on NSCLC cells, following the established protocol [16]. A549 and H358 cells were seeded in a 6‐well cell culture plate at a density of 500 cells per well and allowed to adhere overnight. The cells were then treated with various concentrations of Brazilin (10, 20, 30 μM) or a control. The culture medium was replaced every 2–3 days. After 14 days, the colonies were fixed and analysed for colony formation using crystal violet staining (Solarbio, China).

### 2.6 Transwell Assay

For the Transwell assay, approximately 10,000 A549 and H358 cells were suspended in 200 μL of serum‐free medium and placed into the upper chamber of a Transwell chamber (Corning, USA), with 600 μL of complete medium was added to the lower chamber [15]. After 24 h of incubation, the chamber was removed. Cells were then fixed with 4% paraformaldehyde (PFA) for 30 min and then stained with crystal violet for 15 min. Finally, the chamber was photographed under a microscope, and the stained cells were quantified using ImageJ.

### 2.7 Intracellular ROS Measurement

Intracellular reactive oxygen species (ROS) were measured using the 2′,7′‐Dichlorodihydrofluorescein diacetate (DCFH‐DA) fluorescent probe (#S0033S, Beyotime, Shanghai), as previously reported [17]. A549 and H358 cells were seeded in a 6‐well plate at a density of 2 × 10^5^ cells per well. After allowing the cells to adhere, they were treated with various concentrations of Brazilin for 24 h. Subsequently, the cells were washed three times with serum‐free medium and incubated with 10 μM DCFH‐DA at 37°C in the dark for 30 min. Fluorescence intensity was subsequently measured using microscopy or flow cytometry (Beckman) [18].

### 2.8 Cell Cycle Analysis by Flow Cytometry

A549 and H358 cells were seeded at a cell density of 1 × 10^5^ cells and treated with different doses of Brazilin. After treatment, cells were collected and fixed in ethanol to preserve their DNA content. Fixed cells were stained with PI for DNA content analysis. The stained cells were analysed using flow cytometry (BD FACSAria III) to evaluate their cell cycle distribution. Flow cytometry will provide information on the distribution of cells in different phases of the cell cycle (G0/G1, S, G2/M) based on their DNA content and PI staining intensity, as previously reported [19].

### 2.9 Quantitative Real‐Time PCR

After the Brazilin treatment, the cells were washed twice with PBS. Total RNA was extracted using the Tissue Total RNA Isolation Kit V2 (Vazyme, RC112‐01) and subsequently converted to cDNA with the TransScript Kit (Vazyme, R323‐01). The mRNA expression levels of CCL5, CXCL9, IFN‐β, and CXCL10 in the NSCLC cells treated with Brazilin were quantified using the SYBR Green PCR Kit (Vazyme, Q711‐03). Primers used in this experiment were as previously reported [20].

### 2.10 Flow Cytometry Analysis of PD‐L1, CCL5, and CXCL10

For flow cytometry analysis, 1 × 10^6^ cells were incubated in 100 μL of 0.1% FBS‐PBS with the indicated antibodies (1:100 dilution, BioLegend, San Diego, CA, USA) for 30 min on ice. Following incubation, cells were washed and resuspended in 1 mL of 0.1% FBS‐PBS. The levels of CCL5 and CXCL10 in A549 and H358 cells were measured by flow cytometry. PD‐L1 expression was detected with anti‐human CD274‐PE antibodies, while anti‐APC anti‐human CXCL10 antibodies and PE anti‐human CCL5 antibodies assessed CXCL10 and CCL5 expression, respectively as previously reported [21]. Analysis was performed using a CytoFLEX Flow Cytometer (Beckman Coulter Life Sciences, USA).

### 2.11 Western Blot Analysis

Western blotting was conducted as previously described [22]. After Brazilin treatment, cellular proteins were isolated utilising RIPA buffer, and protein concentrations were measured with the BCA assay. Subsequently, 40 μg of protein from each sample was resolved by 10% SDS‐PAGE gel electrophoresis and transferred to a PVDF membrane (Millipore, Billerica, MA, USA). The membrane was blocked in Tris Buffered Saline with Tween 20 (TBST) solution containing 5% fat‐free milk at room temperature for 1 h. Protein expression levels were assessed using the iBright Imaging System (Invitrogen) and analysed using the Image Lab software (Bio‐Rad).

### 2.12 Determination of Mitochondrial Membrane Potential Levels

Cells were plated onto 6‐well plates at a density of 2.5 × 10^5^ cells per well and allowed to adhere for 24 h. Following adhesion, cells were treated with Brazilin for an additional 24 h. After treatment, cells were washed once with pre‐cooled PBS and incubated with 10 μM 5,5′,6,6′‐Tetrachloro‐1,1′,3,3′‐tetraethyl‐imidacarbocyanine (JC‐1) solution at 37°C in a 5% CO_2_ environment for 30 min. The stained cells were collected by centrifugation at 1200 rpm for 5 min, after which the dye was removed, and the cells were resuspended in PBS. Changes in mitochondrial membrane potential (MMP) in Brazilin‐treated cells were quantified using BD Aria III flow cytometry at an excitation wavelength of 488 nm as previously reported [16]. Red JC‐1 aggregates, and green JC‐1 monomers were detected in the FL2 and FL1 channels, respectively. All analyses were performed using FlowJo software v10.

### 2.13 Statistical Analysis

Data analysis was performed using GraphPad Prism 9.0, with results presented as means ± SD from three independent experiments. Unpaired Student's *t*‐tests were used to compare the means of two groups, while one‐way ANOVA was employed for comparisons among multiple groups. Statistically significant was set as *p < 0.05, **p < 0.01, ***p < 0.001, and ****p < 0.0001.

## 3 Results

### 3.1 Brazilin Effectively Inhibited NSCLC Cell Proliferation In Vitro

**FIGURE 1:**
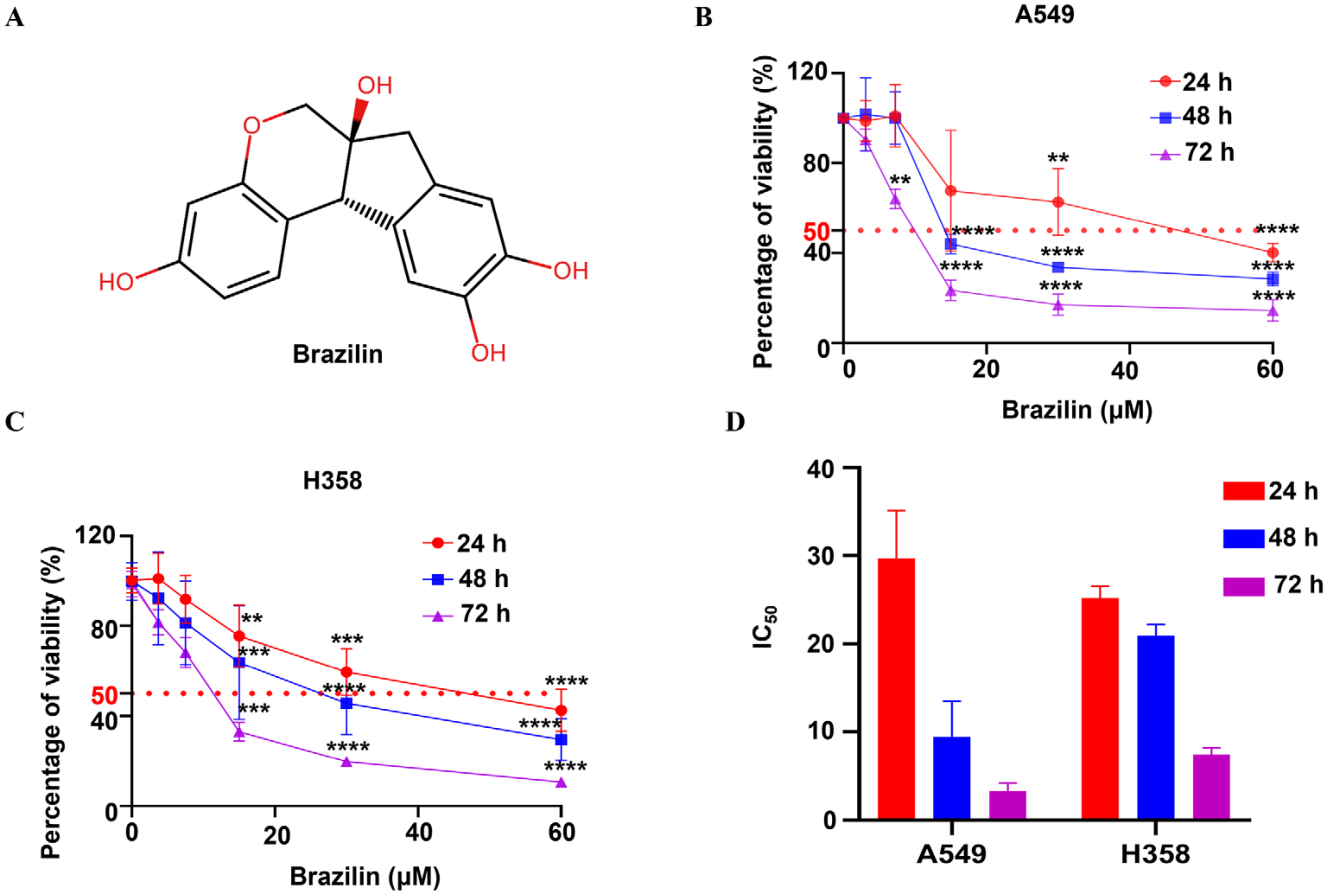
Effect of Brazilin on NSCLC cells in vitro. (A) The structure composition of Brazilin. (B, C) Cell viability of different NSCLC cells incubated with the indicated doses of Brazilin (3.125, 7.25, 15, 30, 60 μM) for 24, 48, or 72 h, and cell viability was detected by CCK8 assay. (D) IC50 values of Brazilin on the A549 and H358 cells after treatment for 24, 48, or 72 h. Statistical significance was denoted as: *p < 0.05, **p < 0.01, ***p < 0.001, ****p < 0.0001.

We investigated the effects of Brazilin on cell growth using two NSCLC cell lines: A549 and H358, both of which carry KRAS mutations. After incubating the cells with Brazilin for 24, 48, and 72 h, we assessed cell viability using the CCK8 method. The chemical composition of Brazilin is shown in Figure 1A. As illustrated in Figure 1B,C, Brazilin significantly reduced the viability of A549 and H358 cells in a concentration‐ and time‐dependent manner. The IC_50_ values were 29.72 μM and 25.21 μM at 24 h, 9.38 μM and 20.89 μM at 48 h, and 3.2 μM and 7.46 μM at 72 h, respectively (Figure 1D). These findings indicate that Brazilin may serve as a promising agent for inhibiting the proliferation of NSCLC cells.

### 3.2 Brazilin Inhibited Proliferation and Invasion of NSCLC Cells

**FIGURE 2:**
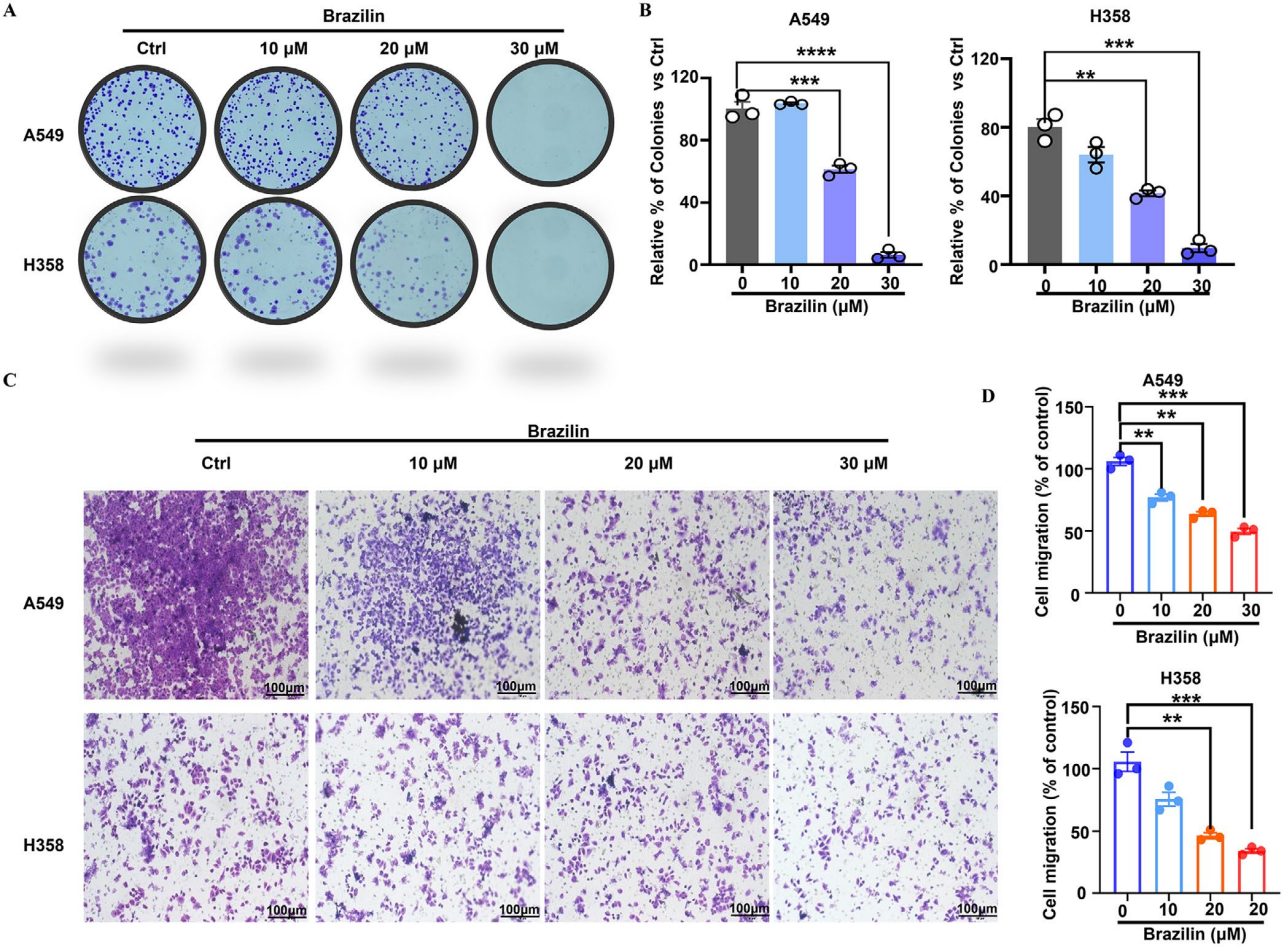
Brazilin inhibited proliferation and invasion of non‐small cell lung cancer cells. (A, B) The colony‐formation assays were performed to evaluate the effects of Brazilin treatment (10, 20, 30 μM) on the numbers of colonies in A549 and H358 cells. Subsequently, the invasion ability of A549 and H358 cells was assessed using crystal violet staining and microscopy (original magnification, 100 μm). Transwell assays revealed a significant reduction in cell invasion after Brazilin treatment (10, 20, 30 μM). Images of cell invasion in each group were captured, and a histogram comparing the number of invading cells across the four groups was presented. Statistical significance was denoted as: *p < 0.05, **p < 0.01, ***p < 0.001, ****p < 0.0001.

Colony formation assays are crucial for evaluating the proliferative capacity of tumour cells [23]. In our study, we employed this technique to examine the effects of Brazilin on the proliferation of NSCLC cells. Our results indicated that Brazilin treatment significantly decreased the colony formation ability of A549 and H358 cells in a concentration‐dependent manner, as shown by the notable reduction in the number of colonies formed (Figure 2A,B). Additionally, we assessed the invasion capabilities of these cells and found that Brazilin also diminished cell invasion in a concentration‐dependent manner (Figure 2C,D). Collectively, these findings demonstrate that Brazilin effectively inhibits both the proliferation and invasion of NSCLC cells, underscoring its potential as a therapeutic agent for NSCLC.

### 3.3 Brazilin Induced Cell Apoptosis in NSCLC Cells

**FIGURE 3:**
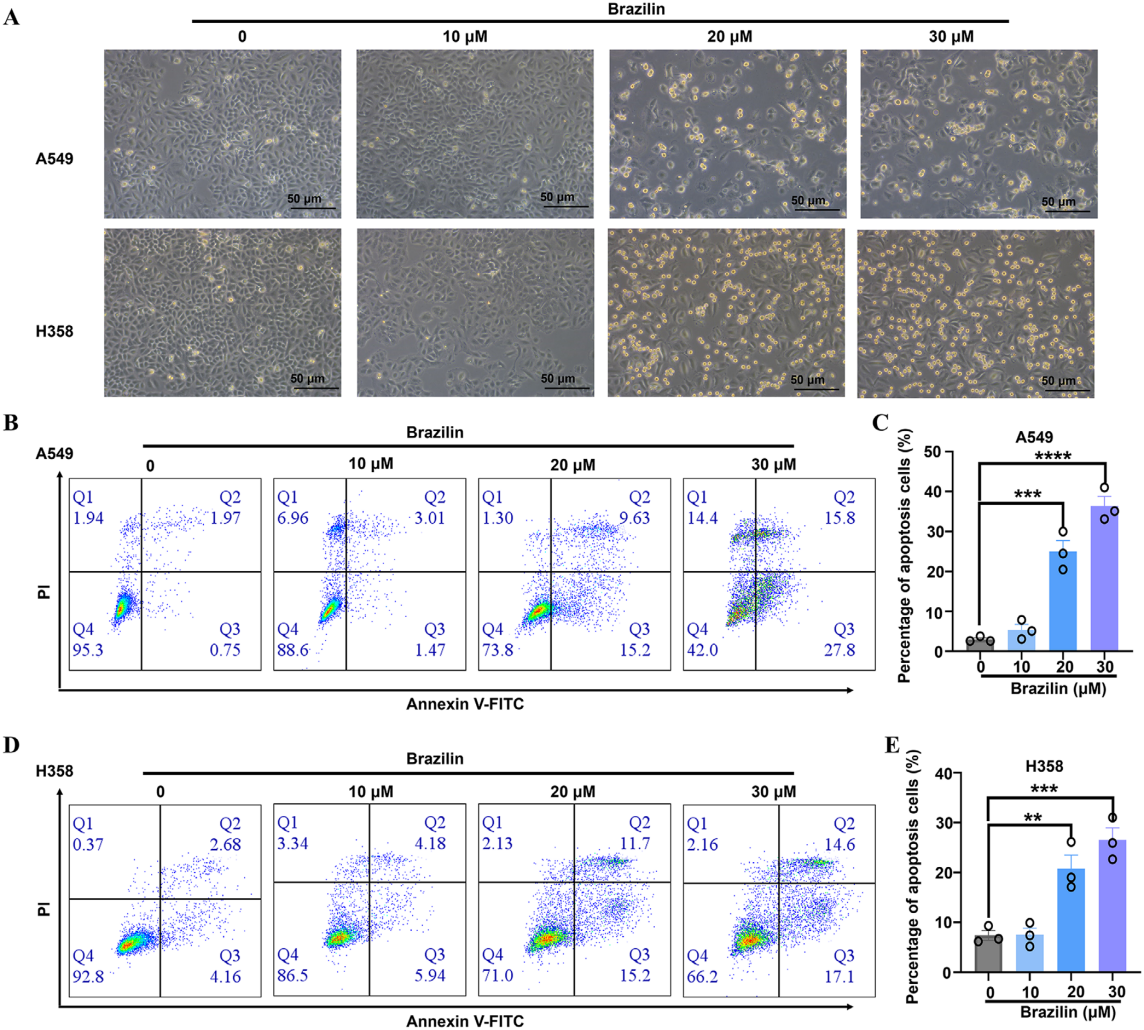
Impact of brazilin on apoptosis in NSCLC cells. (A) The viability of A549 and H358 cells after treatment with Brazilin. (B, C) A549 cells were treated with Brazilin at 10, 20, and 30 μM for 24 h. Cell apoptosis in NSCLC cells was analysed using BD Annexin‐V/PI staining and flow cytometry. (D–E) H358 cells were treated with similarly with Brazilin. Data were presented as the mean ± SD from three independent experiments. Statistical significance was denoted as: *p < 0.05, **p < 0.01, ***p < 0.001, ****p < 0.0001.

To investigate Brazilin's ability to inhibit the growth of A549 and H358 cells through apoptosis, we employed flow cytometry to assess the percentage of apoptotic cells after a 24‐h treatment with Brazilin at concentrations of 0, 10, 20, and 30 μM. Notably, the results indicated a decrease in viable cell counts for both A549 and H358 following Brazilin treatment (Figure 3A), indicating its effect on cell viability. Furthermore, Brazilin induced a concentration‐dependent increase in apoptosis levels in both A549 and H358 cells, as observed in Figure 3B–E. Overall, our results suggest that Brazilin promotes apoptosis in NSCLC cells.

### 3.4 Brazilin Induces G2 Phase Arrest in NSCLC Cells

**FIGURE 4:**
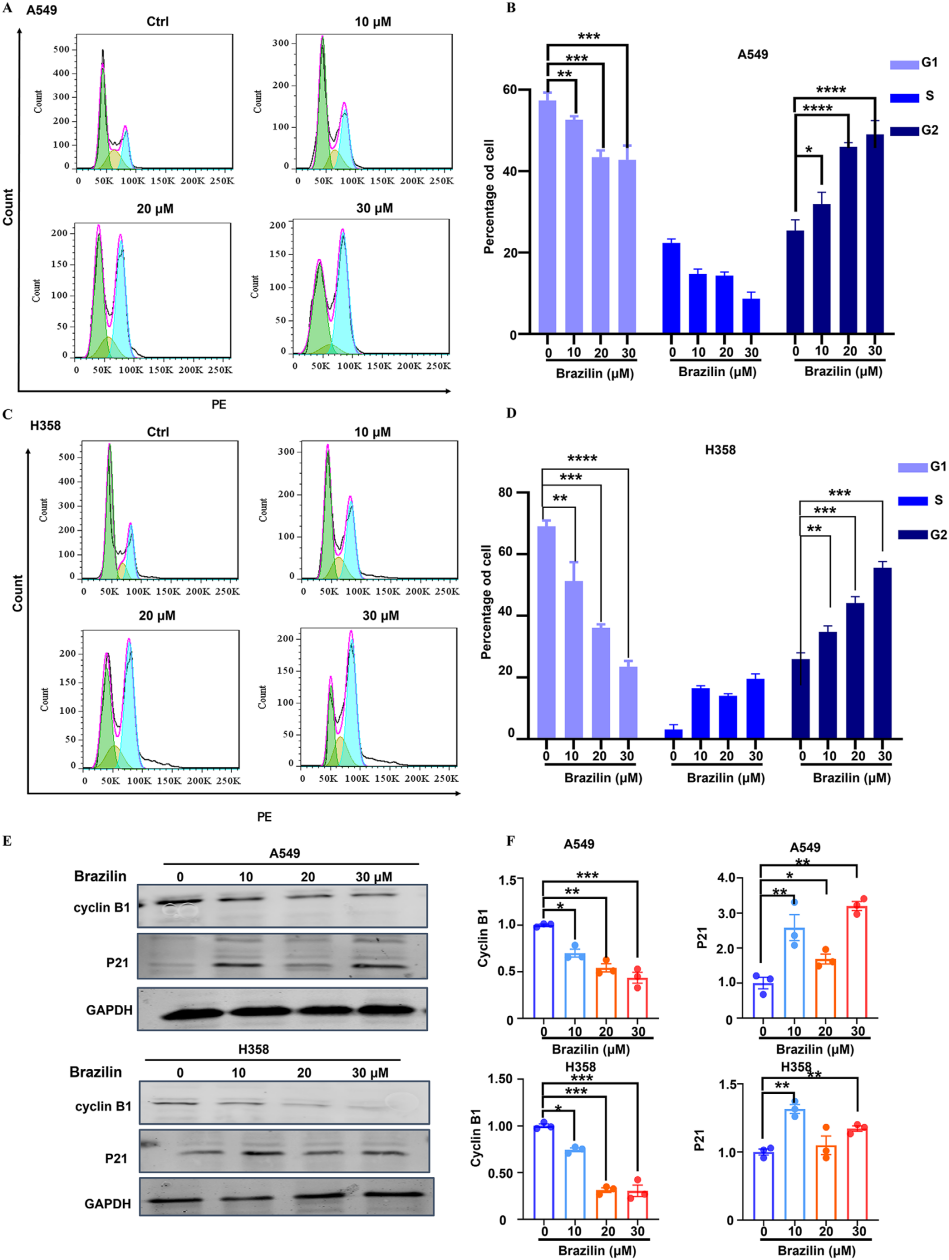
Brazilin significantly arrested the G2 phase of H358 and A549 cell lines. (A–D) Effect of Brazilin on cell cycle distribution of A549 and H358 cells. (E, F) Effect of Brazilin on protein levels of markers of the cell cycle. A549 and H358 cells were treated with escalating concentrations of Brazilin for 24 h. Western blot assays were conducted using specific antibodies against CyclinB1, P21, and GAPDH. Data were presented as the mean ± SD from three independent experiments. Statistical significance was denoted as: *p < 0.05, **p < 0.01, ***p < 0.001, ****p < 0.0001.

To investigate the inhibitory effects of Brazilin on cell growth, we performed a cell cycle analysis using flow cytometry. A549 and H358 cells were treated with varying concentrations of Brazilin (0, 10, 20, and 30 μM) for 24 h. We examined the distribution of cells across different phases of the cell cycle and found that Brazilin treatment significantly reduced cell growth, leading to G2/M phase arrest (Figure 4A–D). To further elucidate the molecular pathways involved in Brazilin‐induced cell cycle arrest, we conducted Western blot analysis. This analysis revealed an increase in P21 protein expression in Brazilin‐treated cells, while Cyclin B1 expression—a key regulator of the cell cycle—decreased during the G2 phase (Figure 4E,F). These findings support the conclusion that Brazilin induces G2 phase arrest in NSCLC cells.

### 3.5 Brazilin‐Induced Mitochondrial Dysfunction and ROS Production in NSCLC Cells

**FIGURE 5:**
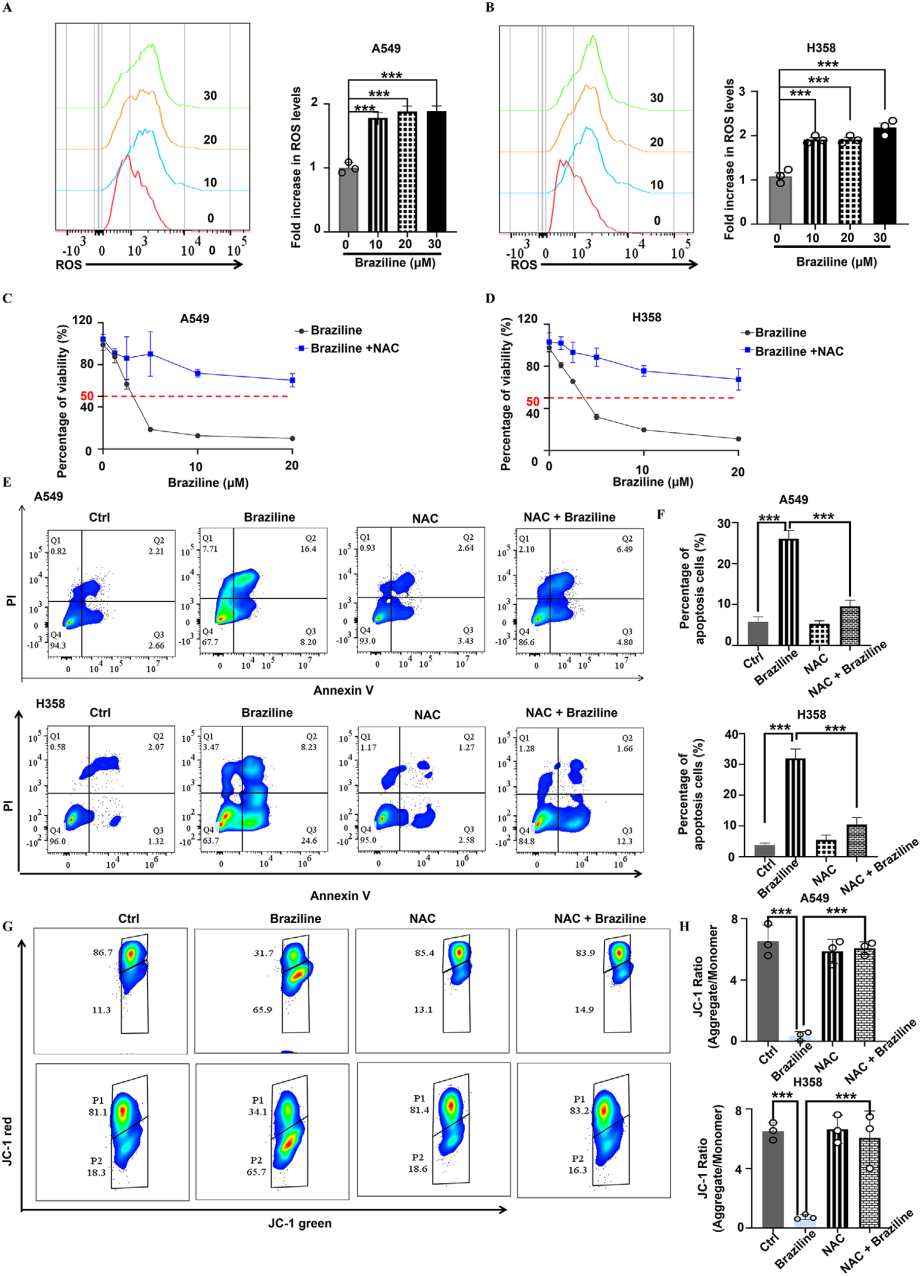
Brazilin induces mitochondrial dysfunction and ROS production in NSCLC cells. (A, B) A549 and H358 cells were treated with Brazilin for 24 h, and ROS levels were quantified using DCFDA staining followed by flow cytometry analysis. (C, D) A549 and H358 cell lines were pre‐incubated with 5 mM N‐acetylcysteine (NAC) for 1 h prior to Brazilin treatment for 24 h, and cell proliferation was assessed using the CCK8 assay. (E, F) Following the same pre‐incubation, A549 and H358 cell apoptosis was evaluated with flow cytometry. (G, H) Mitochondrial membrane potential (MMP) levels were measured using JC‐1 staining and analysed by flow cytometry after pre‐incubation with 5 mM NAC for 1 h, followed by Brazilin treatment for 24 h. Data are expressed as mean ± SD from three independent experiments. *p < 0.05, **p < 0.01, ***p < 0.001, and ****p < 0.0001.

ROS are highly reactive molecules that play a critical role in regulating important signalling pathways in cancer therapy [24]. Dan He et al. demonstrated that Brazilin inhibits breast cancer cells by inducing ROS generation and promoting ferroptosis [25]. To assess whether Brazilin could elevate ROS levels in A549 and H358 cells, we treated these cells with Brazilin and incubated them with 2′,7′‐Dichlorofluorescein diacetate (DCFH2‐DA), a fluorescent probe specifically designed for ROS detection [26]. Flow cytometry analyses revealed a significant increase in ROS levels in both A549 and H358 cells following Brazilin treatment (Figure 5A,B). To elucidate the relationship between ROS and the suppression of cell growth by Brazilin, we conducted CCK‐8 assays. We utilised the ROS scavenger NAC to assess its influence on Brazilin‐induced proliferation inhibition. Pre‐treatment with NAC effectively countered Brazilin's inhibitory effects on cell growth in both A549 and H358 cells (Figure 5C,D). Notably, NAC pre‐treatment significantly reduced Brazilin‐induced apoptosis (Figure 5E,F).

Mitochondria are essential for initiating apoptosis, and a loss of mitochondrial membrane potential (MMP) serves as an indicator of this process [27]. To evaluate MMP in NSCLC cell lines, we employed 5,5′,6,6′‐Tetrachloro‐1,1′,3,3′‐tetraethyl‐imidacarbocyanine (JC‐1) staining [28]. As shown in Figure 5G,H, the reduction in MMP indicates an early phase of apoptosis and signifies mitochondrial dysfunction. Additionally, our JC‐1 staining revealed that Brazilin reduced MMP in NSCLC cell lines, and interestingly, NAC was able to reverse Brazilin‐induced MMP loss (Figure 5G,H). Collectively, our findings suggest that ROS play a crucial role in Brazilin‐induced cell death and growth inhibition in NSCLC cells.

### 3.6 Brazilian Significantly Activated STING Pathway in NSCLC Cells

**FIGURE 6:**
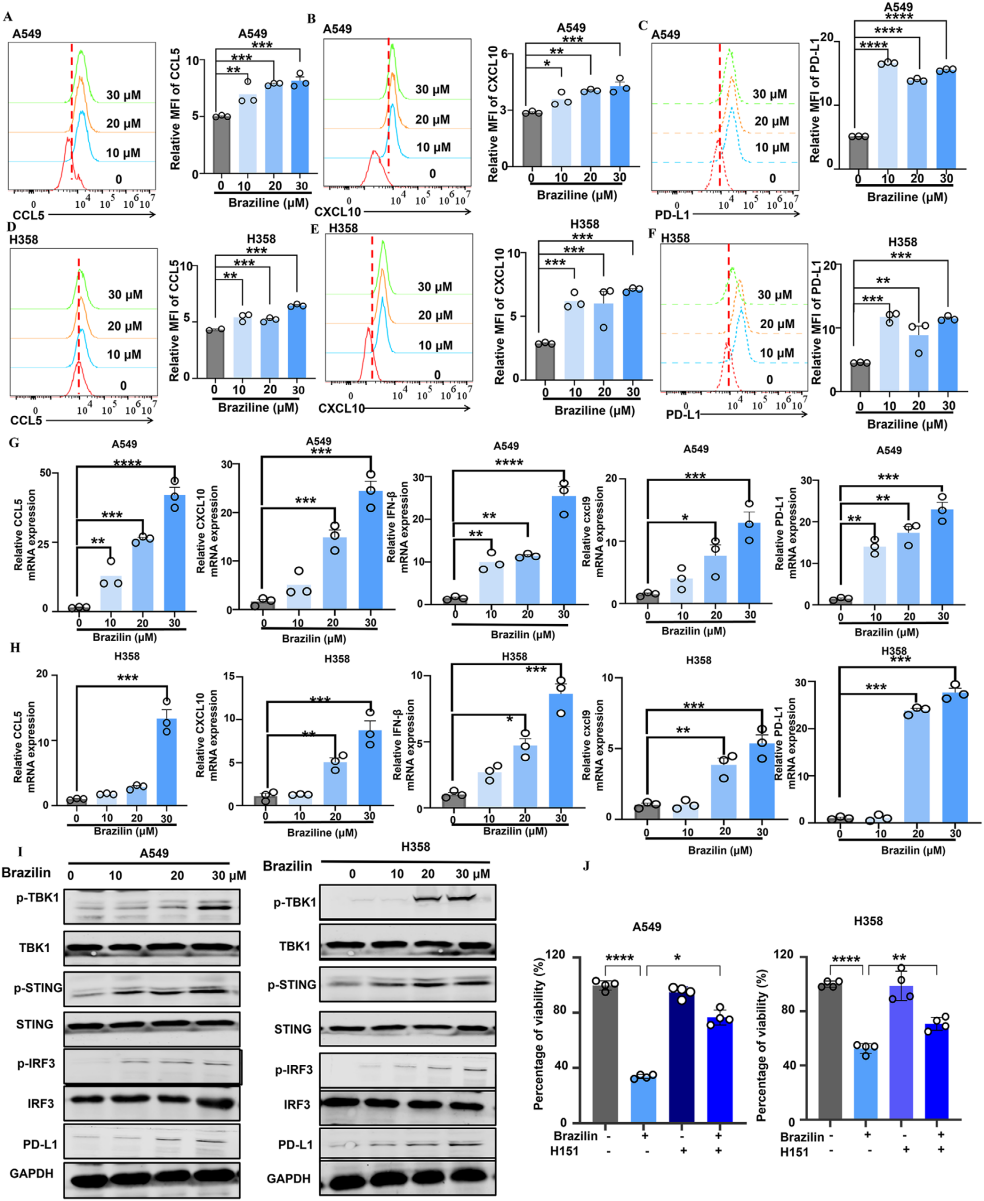
Brazilin significantly activated the STING pathway in NSCLC. (A–D) A549 and H358 cells were treated with Brazilin for 24 h, and CCL5, CXCL10 and PD‐L1 were examined with flow cytometry. (G, H) A549 and H358 cells were treated with Brazilin for 24 h, and the mRNA expression of CCL5, CXCL10, IFN‐β, PD‐L1, and CXCL9 was determined using quantitative RT‐PCR. (I) A549 and H358 cells were pretreated with Brazilin for 24 h. Western blot was performed to determine the protein expression of p‐STING, STING, p‐TBK1, TBK1, p‐IRF3, IRF3, and GAPDH. (J) A549 and H358 cell lines were pre‐incubated with H‐151 for 1 h before treatment with Brazilin for 24 h. The cell proliferation of A549 and H358 cell lines was detected by CCK8 assay. Data were presented as the mean ± SD from three independent experiments. Data are expressed as mean ± SD from three independent experiments. *p < 0.05, **p < 0.01, ***p < 0.001, and ****p < 0.0001.

The STING pathway plays a vital role in the tumour microenvironment by influencing both cell proliferation and anti‐tumour immunity [29]. Activation of this pathway results in the release of chemokines such as CCL5, CXCL9, and CXCL10, which recruit immune cells—including CD8^+^ T lymphocytes, macrophages, and dendritic cells—into the tumour microenvironment [6]. These chemokines have been linked to enhanced efficacy of intensive tumour treatments [30]. Our experiments demonstrated that Brazilin significantly increased the expression of CCL5, programmed cell death ligand 1 (PD‐L1), and CXCL10 in A549 and H358 cells, as confirmed by flow cytometry data (Figure 6A–F). To further explore the impact of Brazilin on chemokine activation, we performed qPCR to quantify mRNA expression levels of CCL5, CCL9, IFN‐β, and CXCL10. The results indicated that mRNA levels of PD‐L1, CCL5, CXCL9, CXCL10, and IFN‐β were significantly elevated in A549 and H358 cells treated with Brazilin (Figure 6G,H). As shown in Figure 5I, Brazilin enhances the phosphorylation of STING, TBK1, and IRF3 in A549 and H358 cells (Figure 6I). Furthermore, STING antagonist (H‐151) was found to counteract Brazilin's inhibitory effect on cell proliferation in both A549 and H358 cells (Figure 6J). Overall, our findings suggest that Brazilin effectively activates the STING pathway in NSCLC, leading to the upregulation of immunoregulatory factors such as chemokines.

## 4 Discussion

In this study, we demonstrated that Brazilin effectively inhibits cell proliferation in NSCLC cells with KRAS mutations in a concentration‐dependent manner, highlighting its potential as a therapeutic agent against these malignancies. Additionally, our findings indicated that Brazilin activates the STING pathway, leading to the production of chemokines, including CCL5 and CXCL10, in NSCLC cells. Moreover, the STING antagonist H‐151 counteracts Brazilin's inhibitory effects on cell proliferation in both A549 and H358 cells.

Lung cancer remains the most prevalent and deadly cancer globally, presenting significant treatment challenges and a poor prognosis [1]. Current clinical management for advanced NSCLC includes chemotherapy, targeted therapy, and immunotherapy [31]. However, these strategies often have limitations: chemotherapy can result in severe side effects and the development of drug resistance, while the efficacy of targeted therapies is frequently reduced in patients with KRAS mutations [32]. KRAS mutations, which are found in approximately 25%–30% of NSCLC cases, compound these challenges, as they are commonly associated with chemotherapy resistance and the absence of effective targeted therapies, leading to poorer patient outcomes [33]. Treatment guidelines from the National Comprehensive Cancer Network (NCCN) underscore the urgent need for innovative therapeutic strategies to tackle these issues [34]. Patients with KRAS mutations frequently demonstrate inadequate treatment responses and a rapid onset of resistance [35], highlighting the necessity for novel agents that are both effective and selective in treating NSCLC. Traditional Chinese medicine has gained increased attention for its robust therapeutic properties and potential in cancer treatment [36, 37].

In this study, we selected the A549 and H358 cell lines, both of which harbour distinct KRAS mutations (G12S and G12C, respectively), to evaluate Brazilin's efficacy against KRAS‐driven tumours. The choice of these cell lines, which represent prevalent KRAS variants, allows for a comprehensive assessment of Brazilin's effects. Notably, A549 cells possess a TP53 mutation, while H358 cells retain wild‐type TP53, thereby minimising confounding factors related to genetic variability and enhancing our investigation of Brazilin's mechanisms [38]. A 2021 study published in Cancer Research highlights the significance of rational cell line selection strategies, further validating the robustness of our experimental design [39].

Brazilin, a natural plant isoflavonoid derived from the *Haematoxilum brasiletto*, has exhibited promising anticancer properties across various cancer types [12]. Previous studies have indicated that Brazilin exhibits anticancer effects in multiple contexts, including lung cancer [40]. For instance, Lorena Cayetano‐Salazar et al. reported that Brazilin suppresses breast cancer cells by upregulating E‐cadherin while downregulating vimentin and twist [41]. Additionally, Dan He et al. discovered that Brazilin induces ferroptosis in breast cancer cells through the p53/SLC7A11/GPX4 signalling pathway [25]. Furthermore, Xihua Yang et al. demonstrated that Brazilin Inhibits the invasion and metastasis of breast cancer [42]. Suyatmi et al. reported that Brazilin enhances apoptosis in A549 cells [43]. While the specific mechanisms of action of Brazilin in NSCLC require further investigation, its diverse biological activities have garnered significant attention. Our findings support the efficacy of Brazilin inhibiting cell proliferation and inducing G2 phase arrest in A549 and H358 cells.

NSCLC cells exhibit elevated mitochondrial bioenergetics, which correlate with altered metabolic pathways. Certain chemotherapeutic agents operate by increasing ROS levels and disrupting mitochondrial membrane integrity, potentially leading to DNA damage and inhibiting cancer progression [44]. Elevated ROS levels also induce oxidative stress, which can promote cellular senescence or apoptosis. Consequently, therapeutic strategies that elevate ROS levels could selectively target various cancer cell types [45]. In our study, we demonstrated that Brazilin induces mitochondrial dysfunction and ROS production in NSCLC cells by reduced MMP in NSCLC cell lines.

STING plays a crucial role in the immune response by recognising cytosolic DNA; upon activation, it triggers the production of type I interferons, thereby enhancing anti‐tumour immunity, upon activation, it triggers the production of type I interferons, thereby enhancing anti‐tumour immunity [6]. This immune response can directly inhibit tumour cell proliferation and promote cell death. Additionally, STING activation facilitates the recruitment and activation of immune cells, such as T cells, augmenting their ability to target cancer cells [46]. Recent studies have investigated STING agonists as potential cancer therapies, seeking to harness this pathway to combat malignancies [47]. However, in KRAS‐mutant tumours, the STING pathway is frequently suppressed. For example, one study identified the loss of STING expression as a poor prognostic indicator in NSCLC with KRAS mutations [48]. This observation led us to hypothesise that activating the STING pathway could counteract the immunosuppressive environment associated with KRAS mutations, potentially overcoming existing therapeutic challenges. Our results demonstrated that Brazilin‐induced expression of CXCL10 and CCL5 activates the STING pathway in NSCLC cells [49]. This finding suggests that Brazilin not only inhibits tumour proliferation but also has the potential to enhance the immune response against KRAS‐mutant tumours.

Notably, the upregulation of P21, a cyclin‐dependent kinase inhibitor associated with p53 activity, and the downregulation of Cyclin B1 are significant indicators of G2/M checkpoint activation and increased genomic instability [50, 51]. Our data demonstrates that Brazilin induces G2 phase arrest in NSCLC cells, prompting important questions: Is the observed cell cycle arrest causally linked to STING pathway activation? Addressing these inquiries will be pivotal for overcoming the limitations of our study. Increased phosphorylation of TBK1 and IRF3 indicates direct pathway activation and enhances the transcription of type I interferons (IFN‐β) and chemokines (CXCL10 and CCL5), further reinforcing this activation [52]. The use of the H‐151 inhibitor helped corroborate the reversal of Brazilin's antiproliferative effects, underscoring the central role of the STING pathway in this therapeutic context. Thus, Brazilin manifests a dual‐action modality—exhibiting both direct antiproliferative effects and immune modulation—which may enable it to overcome resistance mechanisms that typically hinder the efficacy of single‐pathway activators. This multifaceted approach could pave the way for more effective treatments for NSCLC, particularly in the context of KRAS mutations, where existing therapies often fall short.

This approach represents a promising frontier in immunotherapy, offering enhanced potential for effective cancer treatments. Several natural compounds, including sesquiterpenes and flavonoids, have demonstrated the ability to activate the STING pathway and stimulate immune responses against tumours [53]. These natural products promote the body's recognition and elimination of cancer cells by enhancing the production of type I interferons and other cytokines [54]. In this paper, we found that Brazilin activates the STING/TBK1/IRF3 signalling pathway in A549 and H358 cells. However, the in vitro model used in this study cannot fully replicate the complexity of the tumour microenvironment (TME) and cannot replace the results of in vivo experiments. The implementation of 3D co‐culture models or organoids in future research could enhance the relevance of our findings and provide a more accurate representation of cell interactions within the TME. Additionally, the specificity of H‐151 inhibitors raises questions about their effects on other DNA sensing pathways, such as AIM2. To address this concern, further validation using siRNA knockdown technology would be beneficial. The pharmacokinetic properties of Brazilin, particularly its solubility and bioavailability, also present significant challenges. Future research addressing these issues—such as the adoption of nanodelivery systems or the development of prodrug modifications—could enhance its therapeutic potential and effectiveness.

Moreover, initiating retrospective clinical studies to investigate KRAS and STING expression levels could assist in identifying patient populations that are more likely to respond favourably to Brazilin treatment. Furthermore, it is essential to explore the interactions between Brazilin and immune checkpoint inhibitors, as this could amplify STING pathway activation and its role in the DNA damage response. Such investigations may reveal novel combination strategies that could improve the therapeutic efficacy of treatments for KRAS‐mutant cancers.

## 5 Conclusions

Our findings indicate that Brazilin hinders the growth of NSCLC cells by inducing G2/M phase arrest and subsequent apoptosis. Additionally, Brazilin activates the STING/TBK1/IRF3 pathway. Notably, Brazilin significantly stimulates the STING pathway in NSCLC, triggering the expression of CXCL10, CXCL9, and CCL5, which could help overcome resistance to immunotherapy. Overall, our research positions Brazilin as a promising candidate for NSCLC therapy, addressing significant unmet medical needs associated with KRAS mutations.

## Author Contributions

**Li‐Ping Kang:** conceptualization (equal), data curation (equal), writing – original draft (equal). **Cong Xu:** validation (equal), writing – original draft (equal). **Pan Xu:** data curation (equal), writing – original draft (equal). **Dong‐Hui Huang:** data curation (equal), funding acquisition (equal), investigation (equal), methodology (equal), writing – original draft (equal). **Ze‐Bo Jiang:** conceptualization (equal), data curation (equal), formal analysis (equal), formal analysis (equal), writing – original draft (equal), writing – original draft (equal).

## Ethics Statement

Ethics approval and consent to participate.

## Consent

The authors have nothing to report.

## Conflicts of Interest

The authors declare no conflicts of interest.

## Data Availability

All data generated or analysed during this study are included in this published article.
